# Exploring the Regimes of Particle Behavior upon Impact via the Discrete Element Method

**DOI:** 10.3390/pharmaceutics16060727

**Published:** 2024-05-28

**Authors:** Chanh Nguyen, Jennifer Curtis, Kambiz Salari

**Affiliations:** 1 Department of Chemical Engineering, University of California, Davis, CA 95616, USA; ctmnguyen@ucdavis.edu; 2 Lawrence Livermore National Laboratory, Livermore, CA 94550, USA; salari1@llnl.gov

**Keywords:** particle breakage, particle impact, DEM

## Abstract

Discrete element method simulations are conducted to probe the various regimes of post-impact behavior of particles with solid surfaces. The impacting particles are described as spherical agglomerates consisting of smaller constituent (or primary) particles held together via surface adhesion. Under the influence of a wide range of impact velocities and particle surface energies, five distinct behavioral regimes—rebounding, vibration, fragmentation, pancaking, and shattering—are identified, and force transmission patterns are linked to post-impact behavior. In the rebounding regime, the coefficient of restitution decreases linearly as impact velocity increases and the particle agglomerate experiences compaction. In the fragmentation regime, rebound velocity generally decreases with increasing fragment size. The rebound velocity of fragments decreases with time except for the smallest fragments, which can increase in velocity due to collisions with other fragments of high velocity. Particle breakage in the pancaking regime does not follow common mechanistic models of breakage.

## 1. Introduction

Particle impact with surfaces occurs in many technologies, including pharmaceutical processing, jet printing, cold spraying, and defense applications involving explosives [1,2,3,4,5,6]. Many pharmaceutical powders and tablets are agglomerates of fine particles. In bulk solids handling and processing, it is important to understand the behavior of these materials as they interact with solid surfaces, as possible particle abrasion and breakage can change material functionality and flowability, as well as result in the loss of valuable solid mass.

Experimental studies have been conducted to investigate the impact process [7,8,9]. However, it is experimentally difficult to obtain accurate information during the impact process due to the short impact duration and (often) small particle size. Hence, experimental investigations are mostly restricted to post-impact particle analysis [10,11], although ultrahigh-speed cameras and pulsed lasers have been more recently used [12,13].

Numerical simulation offers the potential to overcome these challenges. One common way to describe particle behavior under stress is through the use of material strength models [14,15,16]. This treatment is typical for particle compression modeling. However, since material strength models are calibrated with experimental data, even within the same range of calibration, significant discrepancies between predictions can exist as different assumptions and levels of correlation with experimental data are taken [17]. Outside of the calibration range, the validity of the models becomes questionable, and simulation results often deviate from experimental observations [18,19].

The discrete element method (DEM) can provide insight into particle impact behavior by representing the particle as an agglomerate of constituent (or primary) spheres. Using DEM, constituent sphere forces, velocities, and positions are described, so information on stress transmission within the particle during and after impact, as well as post-impact regimes of particle behavior, are provided. Such agglomerates of constituent spheres have been generated using 3D printing for particle compression research studies, and experimental measurements of compressive loads show good agreement with DEM simulations [20,21,22,23]. Other DEM simulations of the compression of spherical agglomerates have studied the effect of microstructure on breakage [24], rock and ballast breakage [25,26,27], and calendaring for lithium-ion battery electrodes [28], to name a few.

Predicting particle impact behavior using DEM depends on the forces holding the constituent spheres in the particle agglomerate together. In applications when solid binding agents are used to generate particle agglomerates or for particles of high mechanical strength, bonded particle models, along the lines of the force models of Brown, Chen, and Ooi [29] or Potyondy and Cundall [30], are typically employed within DEM simulations. Examples of this treatment include the breakage of L-threonine crystals [31] and the abrasion of pharmaceutical tablets [32]. In solids handling for biomass processing, constituent particles are often held together in agglomerates via liquid bridging. In this case, agglomerate breakage requires the rupture of liquid bridges, which depends on the surface tension and the amount of surface liquid [33]. 

In many dry powder drug delivery, pharmaceutical, detergent, and food manufacturing applications, constituent particles are held together by adhesive forces, often arising from attractive van der Waals forces (but could arise from other sources). To model particle impact in these situations, adhesive forces between constituent particles are typically described by the Johnson, Kendall, and Roberts (JKR) theory, in which adhesion is characterized by surface energy during particle contact loading and unloading [34,35]. Using this approach, research studies have investigated the impact breakage of spherical mannitol powder [36] and non-spherical carrier particles (mannitol and lactose) for dry powder inhalation applications [37]. 

In this study, we investigate particle agglomerate behavior upon impact with a wall. We also consider the case in which constituent particles are held together by an adhesive force, as in many pharmaceutical applications. However, in previous studies, the range of parameters was more limited and focused on one regime (typically fragmentation) of post-impact behavior. Here, the emphasis is on the spectrum of particle behavior upon impact, and we explore particle rebound, vibration, pancaking, fragmentation, and shattering. We map out the range of post-impact particle behavior, varying both the surface energy and impact velocity over a wide range. Particle coordination number, primary particle percentage, distribution of resulting daughter (fragment) particle size distributions, and particle velocity and displacement are monitored to analyze and characterize particle behavior upon impact. Forces on constituent particles and particle–particle contact forces are examined to probe force transmission and mechanisms of particle breakage. 

## 2. Materials and Methods

### 2.1. DEM Model with Adhesion

Impacting particles are modeled as agglomerates (or meta-particles) consisting of a large number of spherical, cohesive constituent (or primary) particles of equivalent properties. In order to describe constituent particle–particle interactions, as well as particle interactions with solid structures such as a wall, the DEM applies Newton’s second law of motion to each constituent particle with mass *m_i_*, translational velocity ***v****_i_*, moment of inertia *I_i_*, and rotational velocity ***ω****_i_* such that

(1) $$m_{i}\frac{dv_{i}}{dt}={m_{i}g+F}_{ci}+F_{di}$$

(2) $$I_{i}\frac{d\omega_{i}}{dt}=M_{ci}+M_{di}$$

where ***g*** is the gravitational acceleration, and ***F****_ci_*, ***F****_di_*, ***M****_ci_*, and ***M****_di_* are the cohesive contact force, contact damping force, torque resulting from the contact force, and torque resulting from the contact damping force, respectively. By numerically integrating over a small time step, new velocities and positions are computed for each constituent particle. Forces acting on the constituent particles include gravitational, damping, and cohesive contact forces. Cohesive contact force, ***F****_ci_*, consists of the normal cohesive contact force, *P*, and the tangential cohesive contact force, *T*. Cohesive forces resulting from van der Waals attractions in normal contact are calculated based on the Johnson–Kendall–Roberts (JKR) theory [38]. The relationship between normal contact force *P* and relative normal displacement *α* is given as [39]:

(3) $$\frac{\alpha }{\alpha_{F}}=\frac{3\left(\frac{P}{P_{c}}\right)+2\pm 2{\left(1+\frac{P}{P_{c}}\right)}^{1/2}}{3^{2/3}{\left[\frac{P}{P_{c}}+2\pm 2{\left(1+\frac{P}{P_{c}}\right)}^{\frac{1}{2}}\right]}^{1/3}}$$

where $\alpha_{F}$ is the critical normal displacement at which two adhesive spheres are separated while $P_{c}$ is the “pull-off” force with which the two adhesive spheres can be separated, depending on the constituent particle surface energy (γ). Calculation of cohesive effects in the tangential contact force is based on the work of Mindlin and Deresiewicz [40] and Savkoor and Briggs [41]. It is assumed that a peeling mechanism must take place before sliding occurs [42]. During this peeling process, the tangential contact force is

(4) $$T=8G^{*}a\delta$$

where $G^{*}$ is the effective shear modulus and δ is the tangential displacement. The contact radius, a, is defined as:

(5) $$a^{3}=\frac{3R^{*}}{4E^{*}}\left[P+2P_{c}\pm {\left(4PP_{c}+4P_{c}^{2}-\frac{T^{2}E^{*}}{4G^{*}}\right)}^{1/2}\right]$$

where $R^{*}$ is the effective radius and $E^{*}$ is the effective Young’s modulus. The peeling process continues in a stable fashion until the tangential contact force reaches a critical value $T_{c}$, then the contact radius is reduced to

(6) $$a^{3}=\frac{3R^{*}}{4E^{*}}\left(P+2P_{c}\right).$$

There are two sliding criteria. If $P<-0.3\;P_{c}$, then

(7) $$T=\mu \left(P+2P_{c}+2\sqrt{PP_{c}+P_{c}^{2}}\right){\left[\frac{3P+4P_{c}+4\sqrt{PP_{c}+P_{c}^{2}}}{3P+6P_{c}+6\sqrt{PP_{c}+P_{c}^{2}}}\right]}^{3/2}$$

Otherwise, if $P\geq -0.3\;P_{c}$, then

(8) $$T=\mu (P+2P_{c})$$

where *μ* is the coefficient of friction. Rolling friction is not considered in this work. Similarly, the damping force, ***F****_di_* [43], consists of a normal damping force,

(9) $$F_{d}^{n}=2\beta_{c}\sqrt{m^{*}k_{n}}\frac{\Delta \alpha }{\Delta t},$$

and a tangential damping force,

(10) $$F_{d}^{t}=2\beta_{c}\sqrt{m^{*}k_{t}}\frac{\Delta \delta }{\Delta t},$$

where *β_c_* is the contact damping coefficient, m* is the effective constituent particle mass, *k_n_* is the normal contact stiffness, *k_t_* is the tangential contact stiffness, Δ*δ* is the change in tangential displacement, and Δ*t* is the time step. An in-house DEM code was used to conduct the simulations, and a summary of model equations and definitions of the variables are presented in Table 1.

### 2.2. Agglomerate Preparation

The simulation takes place in a cubic domain of approximately 1 mm × 1 mm × 1 mm. First, a spherical region is created to reserve space for agglomerate generation, and 5000 constituent (or primary) spherical particles of the same size and material are generated randomly within the reserved simulation region. Then, a centripetal force with an acceleration magnitude equal to that of gravity is introduced to pack the constituent particles closely together. Once the agglomerate is stable (i.e., constituent particles have negligible motion), a surface energy associated with the cohesive force is introduced and slowly increased to the desired value while the centripetal force is gradually reduced to zero. This surface energy holds the agglomerate together due to cohesive forces once the centripetal force is removed. The formed agglomerates have diameters of about 105 μm and a solid volume fraction of 0.54. In this study, a wide range of surface energies (10^−7^ J/m^2^ to 1 J/m^2^) are considered. The variation in agglomerate solid volume fraction over this range of surface energies is negligible. Values of the parameters utilized in the simulations are given in Table 2. These parameters, except for the varying impact velocity and surface energy, are similar to those employed by Tong et al. [36] for simulating the impact breakage of mannitol powder.

## 3. Results and Discussion

Particle agglomerates move downward and impact a wall normally. Impact velocities varying from 2 × 10^−3^ m/s to 5 m/s and surface energies varying from 10^−7^ J/m^2^ to 1 J/m^2^ are considered. Over this range of impact velocities and surface energies, particle behavior after impact can be classified into five different regimes—rebound, vibration, fragmentation, pancaking, and shattering. The five regimes are classified based on the following criteria. In the particle rebound regime, the agglomerate (or meta-particle) remains totally intact after impact. No constituent or primary particles (either lone primary particles or primary particles in fragments) break off from the agglomerate, and the initial agglomerate possesses a sufficiently high rebound velocity to exit the simulation domain. In the case of the particle vibration regime, the agglomerate remains totally intact, but the rebound velocity is very low. Hence, the particle agglomerate vibrates just above the wall surface and engages in frequent inelastic collisions with the wall. The fragmentation regime takes place when at least one constituent particle breaks away from the initial agglomerate. Some of these constituent (or primary) particles break away as lone primary particles and other primary particles are members of a particle fragment. Hence, both types of particle fracture [1]—abrasion (generation of fines from the initial agglomerate) and fragmentation (break off of particle pieces of intermediate sizes from the initial agglomerate)—are included in this regime. Specifically, the fragmentation regime is defined when the percentage of constituent particles after breakage is less than 90%. The percentage of constituent particles after breakage is the ratio of the number of constituent (or primary) particles that break off from the initial agglomerate (either as lone primary particles or primary particles in fragments) divided by the total number of constituent particles in the initial agglomerate. Since all constituent particles have the same density, this ratio represents the mass percentage of primary particles that no longer are part of the parent particle. The pancaking regime is a subset of the fragmentation regime in the sense that the percentage of constituent particles after breakage is also less than 90%, but the largest post-impact fragment remains on the wall surface. Finally, the classification of the shattering regime is when the percentage of constituent particles after breakage exceeds 90%. 

**Figure 1:**
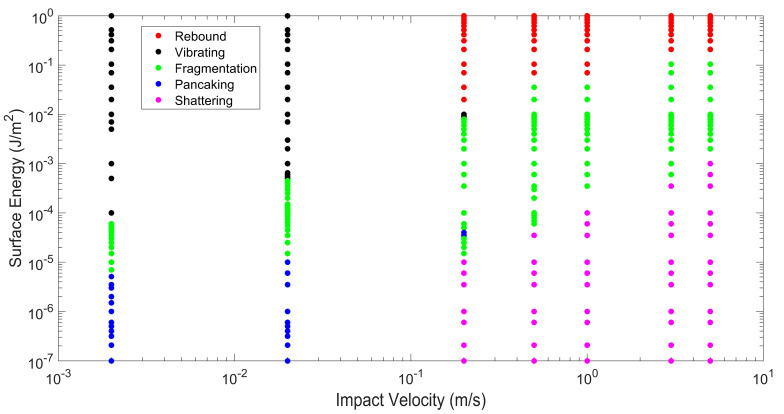
Classification of post-impact particle behavior.

The classification of post-impact particle behavior over a range of surface energies and impact velocities is shown in Figure 1. Generally, at higher impact velocities, as the surface energy increases, the post-impact particle behavior transitions from shattering to fragmentation to rebound. At a higher surface energy, constituent particles are held tightly together, and the agglomerate remains unbroken. When the surface energy is decreased, constituent particles are more loosely held together, and the impact kinetic energy more easily breaks the adhesive bonds between constituent particles. However, when the impact velocity is low, post-impact particle behavior transitions from pancaking to fragmentation to vibration. In this case of low impact velocity and low surface energy, there is not sufficient impact kinetic energy to both break adhesive bonds and propel the largest particle fragment from the wall. Hence, the largest particle fragment remains on the plate. As the surface energy increases at lower impact velocity, fewer particle bonds are broken and there is sufficient remaining impact kinetic energy to propel particle fragments from the wall. When the surface energy is high and the impact velocity is low, no particle bonds are broken, but the initial agglomerate remains intact and engages in repeated impacts with the wall (vibration regime). 

**Figure 2:**
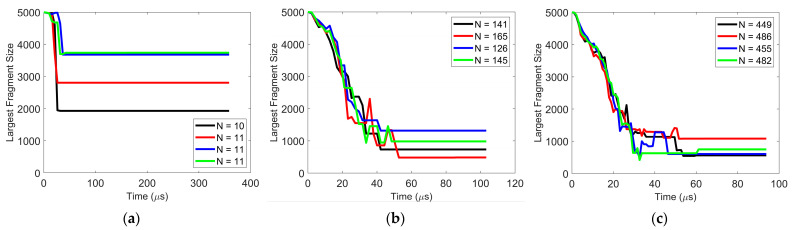
Reproducibility of size of largest fragment after impact with varying impact velocity (**a**) 1 m/s, (**b**) 3 m/s, and (**c**) 5 m/s. Surface energy = 0.02 J/m^2^. The *y*-axis represents the number of constituent (or primary) particles in the largest fragment and N indicates the total number of fragments at steady state.

The reproducibility of these simulations is assessed by having the same initial agglomerate impact the plate on four different random locations on the particle surface. The difference in details of the post-impact particle behavior (velocity, coordination number of agglomerate, primary particle percentage, etc.) is negligible. The only scenario in which there is some level of variability is the case when the particle agglomerate breaks into just a few fragments. Under these conditions, the size of the largest fragment varies depending on the location along the agglomerate surface that first hits the wall. An example of this is shown in Figure 2, in which the *y*-axis represents the number of constituent (or primary) particles in the largest fragment and N indicates the total number of fragments at steady state. At the lower impact velocity of 1 m/s, the agglomerate breaks into approximately 11 fragments, and the size of the largest agglomerate varies. However, as the impact velocity increases and the number of fragments increase, the reproducibility in the size of the largest fragment significantly improves.

### 3.1. Rebound Regime

**Figure 3:**
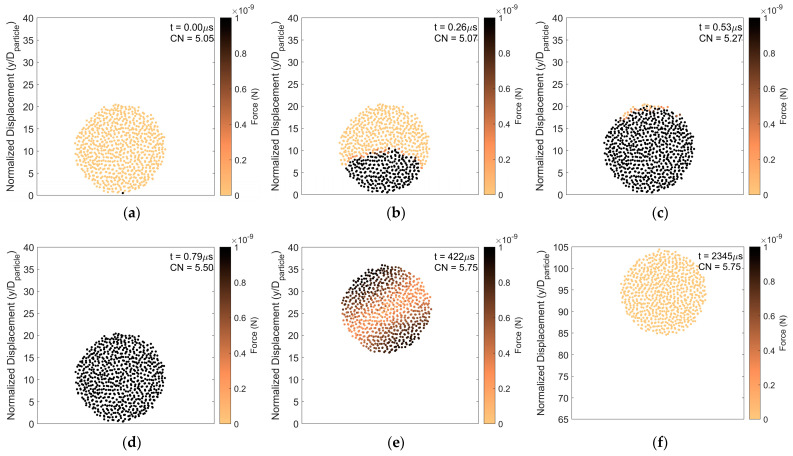
Particle rebound. Force distribution and average coordination number of constituent particles (**a**) 0 μs, (**b**) 0.26 μs, (**c**) 0.53 μs, (**d**) 0.79 μs, (**e**) 422 μs, and (**f**) 2345 μs after initial impact. Surface energy 1 J/m^2^ and impact velocity 0.2 m/s.

Figure 3 presents an example of particle rebound in which the magnitude of the forces on the constituent particles, taken from a center slice of two constituent particle diameters thick, is shown as a function of time after impact. The impact force propagates vertically up through the agglomerate from the initial point of contact. As the force propagates upward, the average coordination number increases as constituent particles are pushed closer together. As time progresses, the force is evenly distributed amongst the constituent particles. However, the post-impact force on the constituent particles at steady state is higher than that before impact. This is consistent with a permanent increase in the average coordination number of the constituent particles in the agglomerate after impact. 

**Figure 4:**
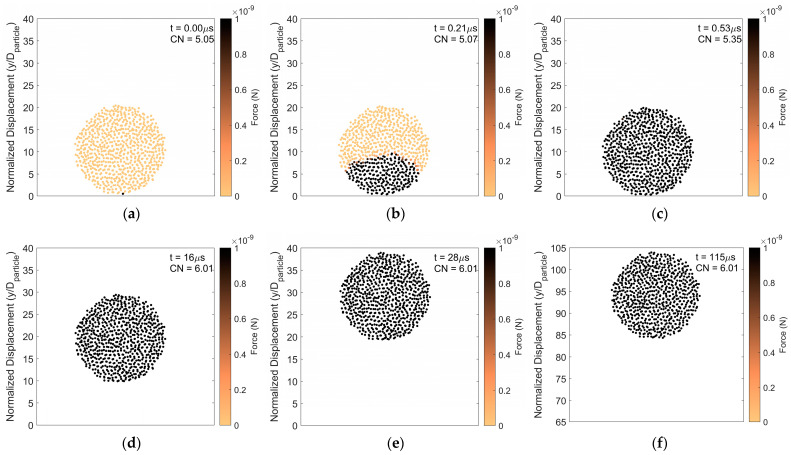
Particle rebound. Force distribution and average coordination number of constituent particles (**a**) 0 μs, (**b**) 0.21 μs, (**c**) 0.53 μs, (**d**) 16 μs, (**e**) 28 μs, and (**f**) 115 μs after initial impact. Surface energy 1 J/m^2^ and impact velocity 5 m/s.

To further demonstrate this, particle rebound analysis at a higher impact velocity but with the same surface energy, is presented in Figure 4. Although the impact velocity is increased, comparing Figure 3 and Figure 4, the impact force advances upward through the agglomerate at the same rate. This suggests that the rate of force propagation does not depend on the magnitude of the impact force but rather depends on how tightly constituent particles are connected. As in the lower impact velocity case, the coordination number increases as the impact force propagates upward and primary particles are pressed together. However, in the case with higher impact velocity, the final steady state force on the constituent particles is substantially increased over its pre-impact magnitude due to the higher impact force. Hence, with increasing impact velocity in the rebound regime, the agglomerate becomes more compacted with a corresponding increase in the average coordination number.

**Figure 5:**
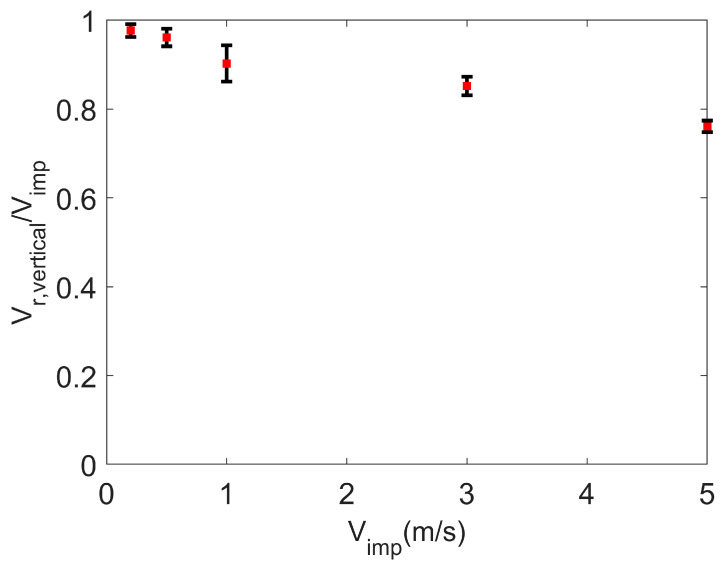
Particle rebound. Coefficient of restitution (ratio of vertical component of rebound velocity to impact velocity) as a function of impact velocity. Surface energy = 1 J/m^2^.

The coefficient of restitution of the agglomerate—the ratio of the vertical rebound velocity to the impact velocity—is shown as a function of impact velocity in Figure 5. The error bars in the figure represent the deviation in rebound velocity for the agglomerate impacting the plate on different points on the agglomerate surface. The coefficient of restitution decreases with increasing velocity, as observed in the experimental measurements of Hassani-Gangaraj et al. [13]. As the impact velocity increases, more of the energy associated with the impact is absorbed in compacting the agglomerate and less is contributing to the particle rebound.

### 3.2. Vibrating Regime

**Figure 6:**
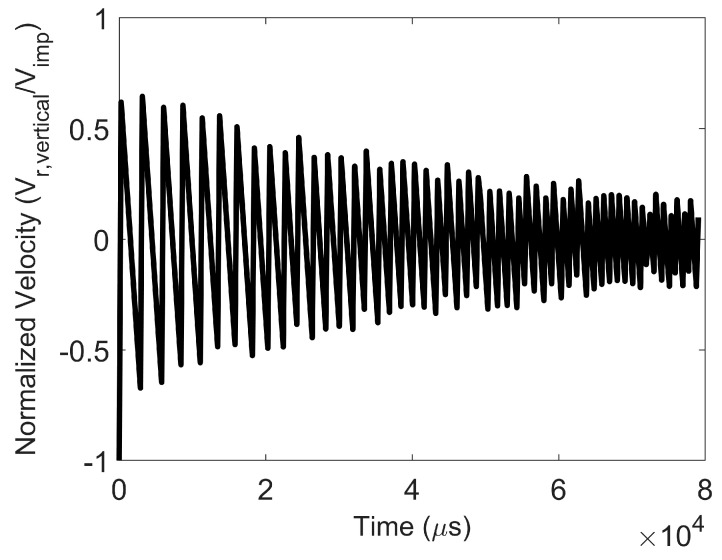
Particle vibration. Evolution of normalized vertical velocity (ratio of vertical component of rebound velocity to impact velocity) with time after impact. Surface energy = 0.02 J/m^2^ and impact velocity 0.02 m/s.

**Figure 7:**
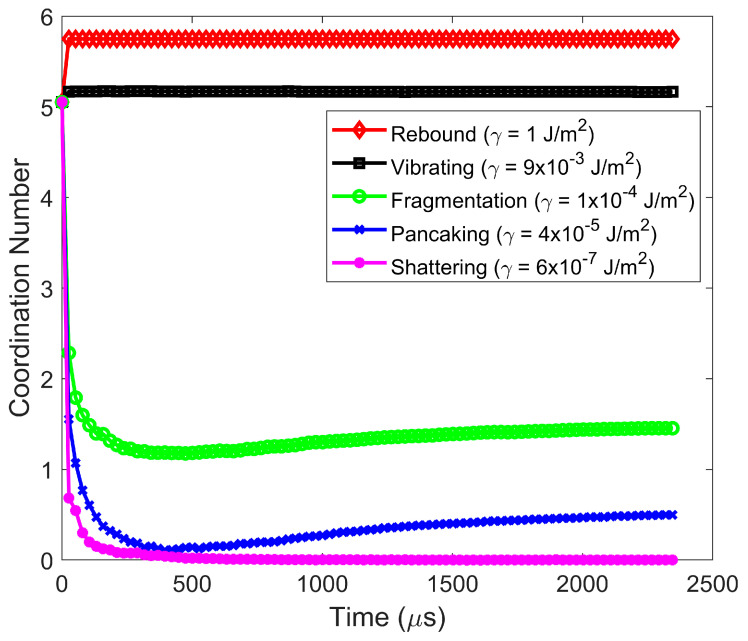
Average coordination number of particles within the agglomerate for the various post-impact regimes. Impact velocity = 0.2 m/s.

The particle vibration regime is present at lower impact velocities. In such cases, as the surface energy is decreased at a fixed impact velocity, the particle remains intact, but the post-impact average coordination number decreases with decreasing surface energy. In addition, the particle rebound velocity decreases to such an extent that it engages in many low-velocity collisions with the wall. In the vibration regime, the rebound velocity decreases rapidly with time due to numerous inelastic collisions with the wall, as shown in Figure 6. In the vibration regime, the average coordination number of particles within the agglomerate (Figure 7) and force on the constituent particles in the agglomerate at steady state post-impact are only minimally higher than those pre-impact.

### 3.3. Fragmentation Regime

**Figure 8:**
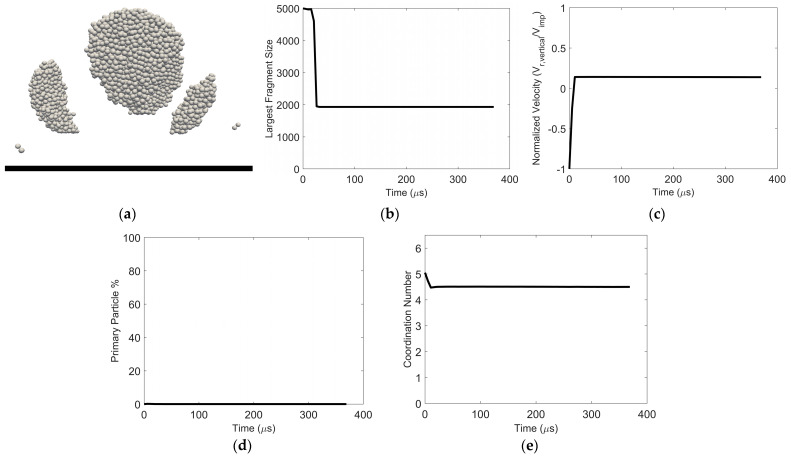
Particle fragmentation (**a**) Image of resulting fragments, (**b**) Largest fragment size, (**c**) Normalized vertical velocity, (**d**) Constituent particle percentage, and (**e**) Average coordination number as a function of time. Impact velocity 1 m/s and surface energy 0.02 J/m^2^.

In the fragmentation regime, the extent of breakage of an agglomerate depends on the impact velocity and surface energy adhering the constituent particles. For a given impact velocity, at high surface energy, the post-impact fragments consist of only a few constituent particles breaking off the agglomerate, as in abrasion. As the surface energy decreases, keeping the impact velocity constant, the fragments become larger but remain low in number, as shown in Figure 8a,b. The percentage of primary particle (single constituent particle) fragments is low (Figure 8d), and the coordination number decreases only slightly from its initial value (Figure 8e). As the surface energy continues to decrease, maintaining the impact velocity, the fragments increase in number and become smaller in size. In all scenarios in the fragmentation regime, the fragments break off and rebound from the wall in different directions. Hence, the average vertical velocity of all the constituent particles contributes to a small average vertical velocity upon rebound (Figure 8c). In addition, some of the impact energy is taken up during the bond-breaking process between constituent particles. 

**Figure 9:**
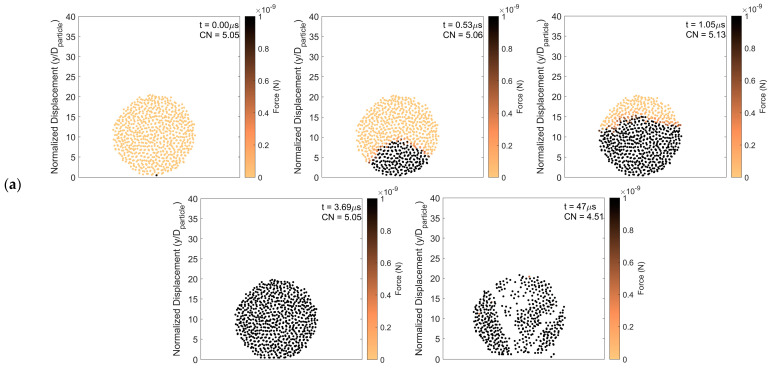

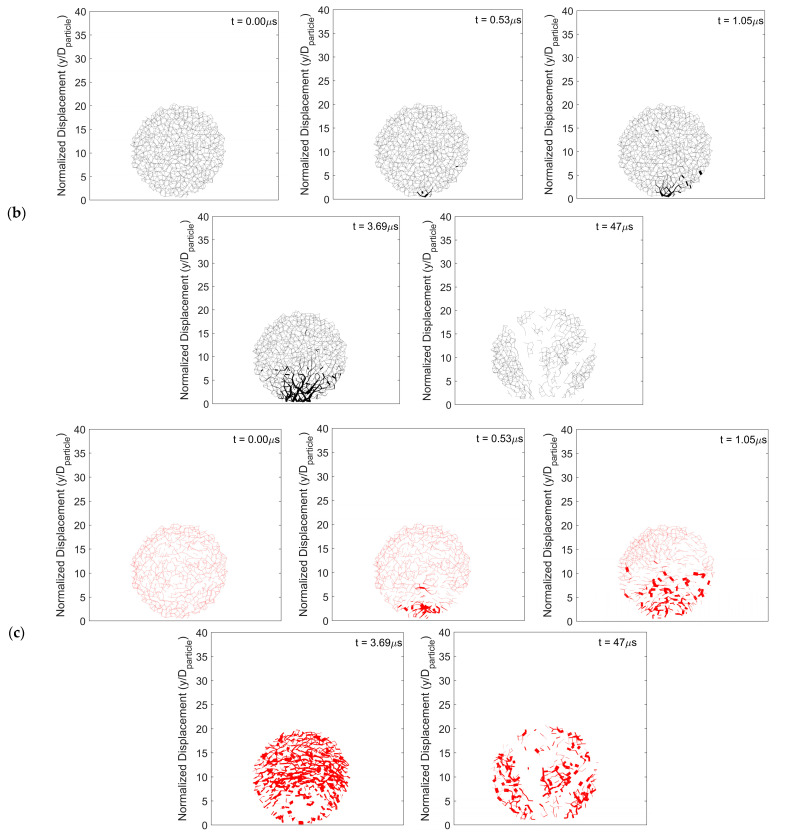
Particle fragmentation. Average coordination number, (**a**) particle force distribution, (**b**) tensile force, and (**c**) compressive force as a function of time. Surface energy = 0.02 J/m^2^ and impact velocity 1 m/s.

Figure 9 presents the force propagation over time and provides insight into particle fragmentation. The simulation data necessary to generate this figure are taken from a center slice of the agglomerate that is two constituent particle diameters thick. Figure 9a represents the total force on each constituent particle. Figure 9b,c show forces between constituent particles, which are represented by lines with increasing levels of darkness corresponding to increasing force. White regions in Figure 9 indicate that there are no forces between constituent particles and, hence, particle breakage is present in those regions. Figure 9a shows that the force on each constituent particle increases with time toward the top of the agglomerate. As in the rebound regime, the coordination number increases as the force propagates since constituent particles are packed more tightly. However, in the fragmentation regime, after the increased force reaches the top of the agglomerate, there is a drop in coordination number corresponding to broken bonds within the particle agglomerate, marking the beginning of the fragmentation process. Figure 9b,c present tensile and compressive forces between constituent particles, respectively. The tensile forces emanating from the point of contact increase with time, shown by the thickening of the line in Figure 9b, and some of these forces exceed the adhesive force. The agglomerate breaks up along these thickened lines. In regions of high tensile force, the compressive force (Figure 9c) is lower.

**Figure 10:**
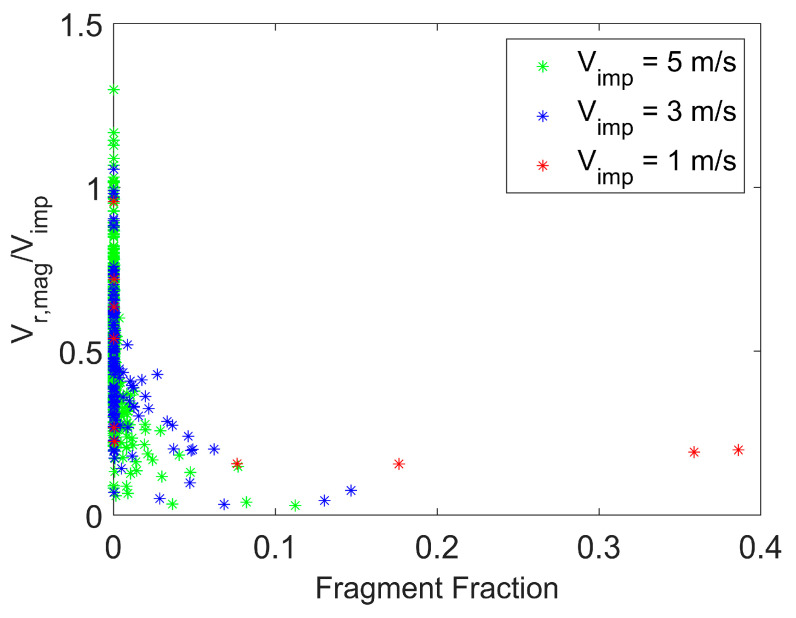
Particle fragmentation. Magnitude of the rebound velocity (considering all three velocity components) scaled by the impact velocity as a function of fragment size for varying impact velocity. Surface energy = 0.02 J/m^2^. Time = 63 μs.

Figure 10 presents the magnitude of the rebound velocity scaled by the impact velocity as a function of fragment size for various impact velocities at a fixed particle surface energy. The magnitude of the rebound velocity in this figure represents the post-impact velocity magnitude considering all three coordinate directions, not just the velocity component perpendicular to the wall. Fragment size is expressed in terms of a “fragment fraction”, defined as the ratio of the number of constituent (or primary) particles in a fragment to the number of constituent particles in the initial particle agglomerate (or meta-particle). Hence, Figure 10 shows the distribution of rebound velocities associated with the distrIbution of daughter particles (with the daughter particles expressed in terms of fragment fraction) for a given impact velocity. The rebound velocities shown in the figure are for a fixed time after agglomerate impact with the wall and after completion of the fragmentation process. As the impact velocity increases, the agglomerate breaks into a larger number of primary particles and smaller fragments, and smaller fragments tend to have a larger rebound velocity than larger fragments. This trend of rebound velocity with fragment size was observed in the experimental measurements of Hassani-Gangaraj et al. [13]. In addition, at higher impact velocity, some of the smallest fragments have rebound velocity magnitudes that exceed the initial impact velocity of the agglomerate. This is due to a large number of post-impact collisions between fragments that lead to additional fragmentation and can also increase the velocity of the smaller fragments.

**Figure 11:**
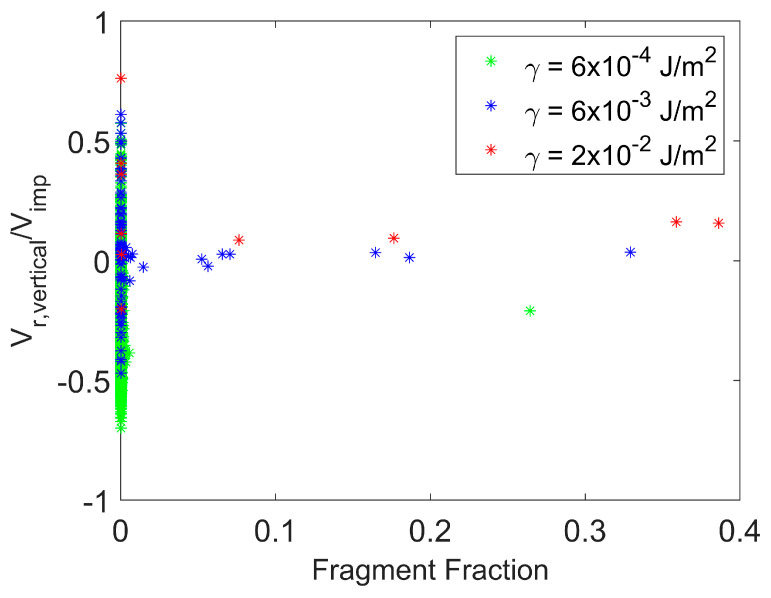
Particle fragmentation. Magnitude of vertical rebound velocity for particle fragments with varying surface energy. Impact velocity = 1 m/s. Time = 47 μs.

Figure 11 presents the magnitude of the vertical rebound velocity scaled by the impact velocity as a function of fragment size for various surface energies at a fixed impact velocity. The magnitude of the rebound velocity in this figure represents the post-impact velocity magnitude considering only the vertical component perpendicular to the wall. The vertical rebound velocities are for a fixed time after agglomerate impact with the wall and after completion of the fragmentation process. The agglomerate breaks into a larger number of smaller fragments as surface energy decreases. Numerous small fragments and primary particles have negative (downward) vertical velocities as a result of collisions with other fragments that propel these particles toward the wall once again. The number of these collisions leading to the downward motion of small fragments increases with decreasing surface energy.

### 3.4. Pancaking Regime

**Figure 12:**
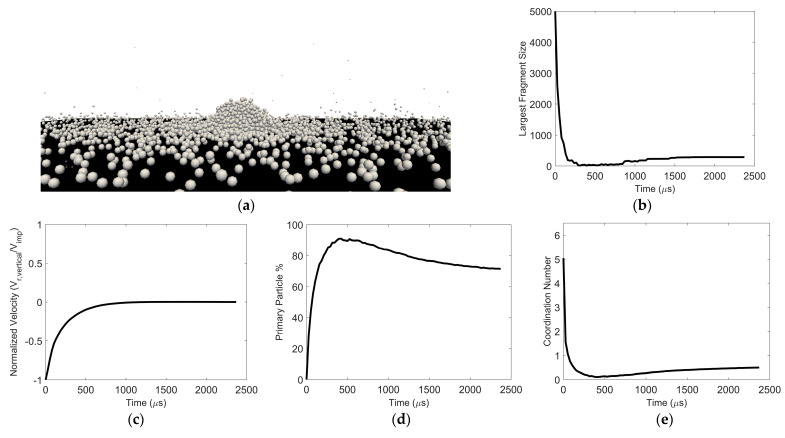
Particle pancaking. (**a**) Image of resulting fragments (**b**) Largest fragment size, (**c**) normalized vertical velocity, (**d**) constituent particle percentage, and (**e**) average coordination number as a function of time. Surface energy = 4 × 10^−5^ J/m^2^ and impact velocity = 0.2 m/s.

As the surface energy is reduced for a fixed (low) impact velocity or as the impact velocity decreases for a fixed surface energy, the average vertical velocity for all the constituent particles approaches zero after post-impact fragmentation. An example of this “pancaking regime” scenario is shown in Figure 12. The largest fragment remains on the wall. The smallest fragments also remain close to the wall, engaging in frequent collisions with the wall and other small fragments near the wall. In fact, these frequent collisions between post-impact fragments close to the surface lead to an increase in the size of some of the fragments with time. This is evident in the non-monotonic dynamic behavior of the size of the largest fragment (Figure 12b), the primary particle percentage (Figure 12d), and the particle coordination number (Figure 12e).

### 3.5. Shattering Regime

At sufficiently high impact velocity for a given surface energy, particle agglomerates will shatter. The post-impact fragments are dominated by individual constituent (primary) particles, and these primary particles are generated at an increased rate with increasing impact velocity. The trajectory of these resulting primary particle fragments depends on the magnitude of the impact velocity. For lower impact velocities, the primary particle fragments remain close to the surface and scatter radially from the point of impact. At higher impact velocity, the primary particle fragments fan out from the point of impact with an increased vertical velocity component.

### 3.6. Comparison with Mechanistic Model

The post-impact particle information generated in this computational study, over a very wide range of impact velocities and particle surface energies, provides a vast amount of simulation data to test the range of applicability of the well-cited mechanistic particle impact model of Moreno-Atanasio and Ghadiri [46]. This mechanistic model for post-impact particle behavior is a scaling relationship developed based on the premise that the energy required to break particle contacts within the agglomerate is proportional to the kinetic energy at impact. The model indicates that the agglomerate damage ratio (defined as the fraction of broken contacts within the agglomerate) scales with a dimensionless quantity ∆ that depends on the impact velocity, surface energy, and other particulate material properties.

(11) $$∆=WeI_{e}^{2/3}=\frac{\rho R^{5/3}E^{2/3}V^{2}}{\gamma^{5/3}}$$

where *We* is the Weber number, *I_e_* is the elastic adhesion index, *ρ* is the constituent particle density, *R* is the constituent particle radius, *E* is the Young’s modulus, *V* is the agglomerate impact velocity, and γ is the surface energy. Hence, the damage ratio data when scaled with the dimensionless quantity ∆ over a wide range of surface energies and impact velocities will all collapse on one master curve describing the post-impact behavior from the rebound regime (damage ratio = 0) to the shattering regime (damage ratio = 1). For a given particulate material, the dimensionless quantity ∆ has large values for high impact velocity and low values of surface energy. Increasing ∆ leads to an increase in the damage ratio.

**Figure 13:**
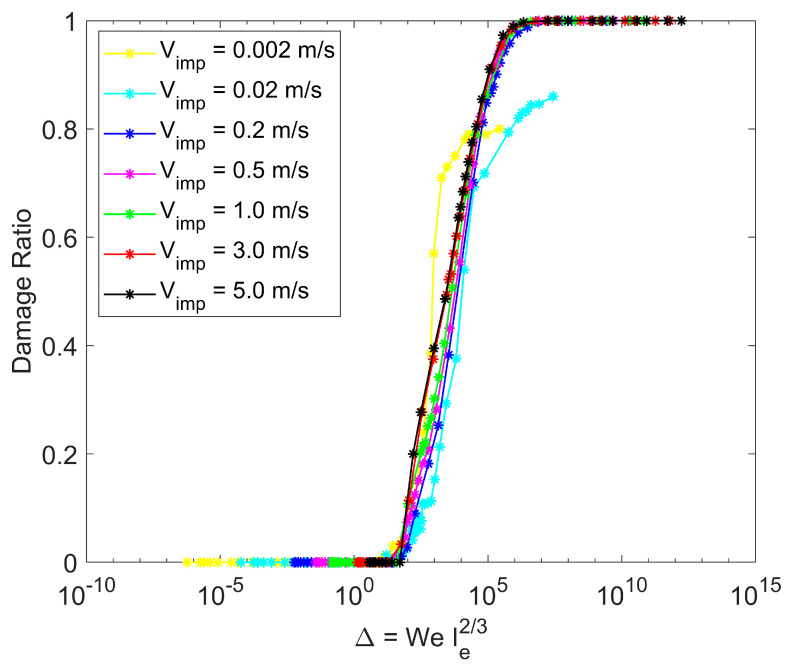
Scaling with the mechanistic model of Moreno-Atanasio and Ghadiri [46].

All the simulation data obtained in this study are scaled in the manner given by this mechanistic model and shown in Figure 13. The predicted damage ratio scales with the dimensionless parameter ∆ for all impact velocities and surface energies except at the lowest impact velocities with low surface energies (larger values of ∆). These conditions correspond to the pancaking regime but represent conditions less likely to be observed in practice.

## 4. Conclusions

This discrete element method study explores the effect of surface energy and impact velocity on the behavior of particle agglomerates impacting a wall. A regime map of post-impact particle behavior is generated based on simulation data over a wide range of surface energies and impact velocities. Five regimes of post-impact behavior are identified: rebound, vibration, fragmentation, pancaking, and shattering. Detailed particle-level information generated from the simulations, such as force propagation, particle coordination number, particle rebound velocity, and fragment size, are used to characterize each regime. Some key findings include:

- Rebound regime: Increasing particle agglomerate compaction and coordination number and decreasing coefficient of restitution with increasing impact velocity.
- Vibration regime: Rapid decay of rebound velocity with time with minimal change in particle agglomerate packing and coordination number.
- Fragmentation regime: Smaller fragments have a larger rebound velocity than larger fragments and the smallest fragments can have rebound velocities which exceed the initial impact velocity. The rebound velocity of the smallest fragments can also be directed towards the wall once again due to collisions between fragments.
- Pancaking regime: Frequent collisions between fragments at or near the wall lead to an increase in the size of some of the fragments with time.
- Shattering regime: The trajectory of the resulting primary particles is radially directed along the plate from the point of impact at lower impact velocities but has a significant vertical component as the impact velocity is increased.

In addition, the wide-ranging simulation results are used to corroborate the mechanistic model of Moreno-Atanasio and Ghadiri [46]. 

It should be noted that while this study focused on the effects of impact velocity and surface energy, the other constituent particle and agglomerate properties (density, Young’s modulus, particle size, agglomerate packing structure) were held constant. The effect of a mixture of different types of particles and their packing arrangement in the agglomerate will be investigated in future work. While the exact magnitudes of impact velocity and surface energy associated with a given post-impact particle behavioral regime depend on the specific particulate system, the regime map presented here provides a guide to the relative relationship between those regimes for particles with different material properties. For example, for a given particulate material, the regime map indicates how the post-impact particle behavior will change with increasing or decreasing impact velocity. In addition, the details of the particle behavior associated with a specific post-impact regime, as outlined in this work, are generally applicable.

An example application of this study is in the operation of a high shear granulator—setting an impeller speed that promotes agglomeration and minimizes granule breakage. The velocity associated with granules interacting with the surfaces of the rotating impeller blade should be sufficiently high to affect good mixing but not so high that the granules break. That is, particle impact with the rotating blade should not result in fragmentation, shattering, or pancaking. Although granules interact with the surface of the rotating impeller at a range of incident angles and only normal impacts are considered here, it is an easy matter to consider the effect of incident angle on post-impact behavior. That said, it has been shown that the normal component of the impact velocity is the dominant factor controlling the breakage of agglomerates [35].

While this study considers a single agglomerate, industrial systems consist of many agglomerates. In this case, particles impact with each other, as well as impact with wall surfaces, so both types of collisions need to be considered. In some cases, however, particle–wall impacts dominate the breakage behavior in an industrial setup. For example, in a vertical-axis mixer, it was found that the largest stresses on the particles occurred on the upstream side of the mixing blade tip and at the bottom wall of the mixer [47]. Nevertheless, the incident angle of those particle–wall impacts will greatly depend on the neighboring particle–particle impacts.

## Figures and Tables

**Table 1:** DEM model equations.

Critical normal displacement	$\alpha_{F}={\left[\frac{3P_{c}^{2}}{16R^{*}E^{*2}}\right]}^{\frac{1}{3}}$	Johnson [39]
Pull-off force	$P_{c}=3\gamma \pi R_{i}$ *R_i_*, radius of constituent particle *i*	Johnson [39]
Critical tangential contact force	$T_{c}=4{\left[\frac{\left(PP_{c}+P_{c}^{2}\right)G^{*}}{E^{*}}\right]}^{\frac{1}{2}}$	Thornton and Yin [42]
Effective radius	$R^{*}=\frac{R_{1}R_{2}}{R_{1}+R_{2}}$	Johnson [39] Thornton and Yin [42]
Effective Young’s modulus	$\frac{1}{E^{*}}=\frac{1-\nu_{1}^{2}}{E_{1}}+\frac{1-\nu_{2}^{2}}{E_{2}}$ *ν_i_,* Poisson’s ratio of particle *i* *E_i_*, Young’s modulus of particle *i*	Johnson [39] Thornton and Yin [42]
Shear modulus	$G_{i}=\frac{E_{i}}{2(1+\nu_{i})}$ *G_i_*, shear modulus of particle *i*	Phani and Sanyal [44]
Effective shear modulus	$\frac{1}{G^{*}}=\frac{2-\nu_{1}}{G_{1}}+\frac{2-\nu_{2}}{G_{2}}$	Johnson [39] Thornton and Yin [42]
Contact damping coefficient	$\beta_{c}=\frac{-ln(e)}{\sqrt{\pi^{2}+\ln^{2}\left(e\right)}}$ *e*, coefficient of normal restitution	Guo et al. [45]
Effective mass	$m^{*}=\frac{m_{1}m_{2}}{m_{1}+m_{2}}=\frac{m}{2}$ *m_i_*, mass of constituent particle *i*	Guo et al. [45]
Normal contact stiffness	$k_{n}=\frac{6E^{*}a\left(1-\sqrt{\frac{3P_{c}R^{*}}{4E^{*}a^{3}}}\right)}{3-\sqrt{\frac{3P_{c}R^{*}}{4E^{*}a^{3}}}}$	Thornton and Yin [42]
Tangential contact stiffness	$k_{t}=8G^{*}a\theta \pm \mu (1-\theta )\frac{\Delta P}{\Delta \delta }$	Thornton and Yin [42]

**Table 2:** Simulation Parameters.

Parameter	Value
Number of constituent particles	5000
Constituent particle diameter, 2R	5 μm
Agglomerate diameter	105 μm
Constituent particle density, *ρ*	1490 kg/m^3^
Young’s modulus, *E*	1 × 10^8^ N/m^2^
Poisson’s ratio, *ν*	0.29
Particle-particle friction coefficient	0.3
Particle-wall friction coefficient	0.3
Particle-particle coefficient of restitution	0.95
Particle-wall coefficient of restitution	0.90
Impact velocity, V_imp_	0.002–5 m/s
Constituent particle surface energy, γ	1 × 10^−7^–1 J/m^2^
Wall surface energy	0 J/m^2^
Time step	1.3 × 10^−8^ s for V_imp_ = 0.002 m/s, 0.02 m/s 5.3 × 10^−9^ s for V_imp_ = 0.2 m/s and 0.5 m/s 1.1 × 10^−9^ s for V_imp_ = 1 m/s, 3 m/s, 5 m/s

## Data Availability

The original contributions presented in the study are included in the article, further inquiries can be directed to the corresponding author.
